# 3D Object Recognition Using Fast Overlapped Block Processing Technique

**DOI:** 10.3390/s22239209

**Published:** 2022-11-26

**Authors:** Basheera M. Mahmmod, Sadiq H. Abdulhussain, Marwah Abdulrazzaq Naser, Muntadher Alsabah, Abir Hussain, Dhiya Al-Jumeily

**Affiliations:** 1 Department of Computer Engineering, University of Baghdad, Al-Jadriya, Baghdad 10071, Iraq; 2 Department of Electronic and Electrical Engineering, University of Sheffield, Sheffield S1 4ET, UK; 3 School of Computer Science and Mathematics, Liverpool John Moores University, Liverpool L3 3AF, UK; 4 Department of Electrical Engineering, University of Sharjah, Sharjah 27272, United Arab Emirates

**Keywords:** 3D recognition, overlapped block processing, features extraction, orthogonal moments, orthogonal polynomials, SVM, Charlier polynomials

## Abstract

Three-dimensional (3D) image and medical image processing, which are considered big data analysis, have attracted significant attention during the last few years. To this end, efficient 3D object recognition techniques could be beneficial to such image and medical image processing. However, to date, most of the proposed methods for 3D object recognition experience major challenges in terms of high computational complexity. This is attributed to the fact that the computational complexity and execution time are increased when the dimensions of the object are increased, which is the case in 3D object recognition. Therefore, finding an efficient method for obtaining high recognition accuracy with low computational complexity is essential. To this end, this paper presents an efficient method for 3D object recognition with low computational complexity. Specifically, the proposed method uses a fast overlapped technique, which deals with higher-order polynomials and high-dimensional objects. The fast overlapped block-processing algorithm reduces the computational complexity of feature extraction. This paper also exploits Charlier polynomials and their moments along with support vector machine (SVM). The evaluation of the presented method is carried out using a well-known dataset, the McGill benchmark dataset. Besides, comparisons are performed with existing 3D object recognition methods. The results show that the proposed 3D object recognition approach achieves high recognition rates under different noisy environments. Furthermore, the results show that the presented method has the potential to mitigate noise distortion and outperforms existing methods in terms of computation time under noise-free and different noisy environments.

## 1. Introduction

Significant effort has been dedicated to developing efficient and reliable remote healthcare systems with the Internet of Things (IoT) applications [1]. This development can be achieved through transmitting efficient and secure medical images and videos of the patients and processing them in a fast and reliable way. To this end, advanced remote monitoring schemes of the patients become essential. In particular, efficient three-dimensional (3D) object recognition techniques could be beneficial to process the images and videos of medical systems. This is due to the ability of object recognition to enable feature extraction, which is essential as it provides unique characteristics that can identify objects [2]. Besides, object recognition is also considered as having the most significant importance in the industrial environment, as it represents each object individually and can distinguish the object [3]. These cues are used to extract discriminative features for accurate recognition [4]. Therefore, there is an increasing interest in object recognition, especially in the fields of machine vision, pattern recognition, and machine learning applications [5,6,7]. Various domains, including facial identification [8], gender description [9], and gesture analysis, among others, use object recognition. Object recognition is also used in object identification, medical diagnosis, security applications, multimedia communication, and computer interface applications [4,10].

### 1.1. Related Works

Object recognition and classification can be considered essential techniques, which are beneficial in various applications such as healthcare systems, pattern recognition, molecular biology, and computer vision [11,12,13,14,15]. To this end, significant research works have been developed for efficient 3D object recognition. Besides, feature extraction for 3D objects is extremely useful for classification [16]. Extensive researches have been carried out to develop 3D object classification methods. Some of these works are based on the principles of moment invariants and 3D moments. To this end, a method of 3D translation, rotation, and scale invariants (TRSI) was developed in [17] from geometric moments and an alternative approach was presented later in [18]. A tensor approach to derive the rotation invariants from the geometric moments was proposed in [19]. Besides, an automatic algorithm was proposed in [20,21] to generate 3D rotation invariants from geometric moments. Recently, a 3D Hahn moments combined with convolutional neural networks (CNN) was proposed in [22] to enhance the 3D object classification. Specifically, the work in [22] proposed a hybrid approach based on combining the 3D discrete Hahn moments and CNN to improve 3D object classification. A multi-layer artificial neural network (ANN) perception approach was proposed in [23] for the classification and recognition of 3D images. In [24], a deep learning approach based on neural network and Racah-based moments was proposed for 3D shape classification. Additionally, in [16], an approach based on the combination of 3D discrete orthogonal moments and deep neural network (DNN) algorithms was proposed to improve the classification accuracy of the 3D object features. In [25], a 3D discrete Krawtchouk moments method was proposed for content-based search and retrieval applications. In [26], a 3D image analysis was considered using Krawtchouk and Tchebichef polynomials, where orthogonal moments were exploited to characterize various types of 2D and 3D images. To this end, orthogonal moments are used in many applications such as image analysis [27,28], face recognition [29], pattern recognition [30,31], steganography [32], image reconstruction [33,34], and medical image analysis [35,36].

The recognition process depends extremely on the feature extraction process, which is used to distinguish between different objects. To this end, the process of object localization and object normalization is considered essential for feature extraction technique [37]. As such, essential issues in object recognition and computer vision applications are the extraction of significant features from objects [38]. Object recognition to date is still a challenging problem that affects pattern recognition. This is because the accuracy of object recognition can be affected by class variations [4,39,40]. In particular, different methods are utilized to extract the features from the images. These methods can be classified as deep-learning-based methods, orthogonal-moment-based methods, and texture-based methods [41,42,43,44,45]. While the recognition accuracy of deep-learning-based methods can be very high, these methods run into a substantial amount of computational complexity, as explained in [46,47,48]. In the orthogonal moment approaches, the features of the object are calculated efficiently through the use of Orthogonal Polynomials (OPs) techniques [49]. Due to their effectiveness, orthogonal moments (OMs) and OPs have been widely exploited in recent years for pattern recognition, form descriptors, and image analysis [50,51]. The OMs-based method gives a powerful capability for evaluating the image components because the image components can be efficiently represented in the transform domain [49]. In many object recognition applications, OMs can be utilized to extract features. It is possible to consider the OMs as a scalar approach that is utilized to define and characterize a function. Such OMs can be used to achieve an effective extraction of the features. The OPs function also contains the coordinates of an image in addition to OMs [52,53]. According to work performed in [44], OMs can be exploited in feature extraction from images with various geometric invariants, including translation, scaling, and rotation. In general, various types of moments can be used for image processing. For instance, due to their simplicity, geometric moments are favored above other types of moments [54]. To depict an image with the least amount of redundancy possible, a Zernike and pseudo-Zernike moments approach was developed in [55]. In [55], a moments-based approach was proposed by exploiting the fractional quaternion for colored image detection [56]. This is because the fractional quaternion, which is considered an opposed approach to integer-order polynomials, can represent functions, according to [44]. Furthermore, the diagnosis of plant diseases has been accomplished using fractional-order moments [57]. In [58], the image analysis used Zernike and Legendre polynomials, which act as the kernel functions for Zernike and Legendre moments, respectively. In particular, the Zernike moments approach has the property of invariance in addition to its capability of image data storing and processing with the least amount of redundancy. However, because the Zernike moments approach focused only on the continuous domain, such an approach would require image coordinate adjustments and transformations for discrete domain [59,60]. To address the challenge of computing continuous moments in image analysis, the discrete OMs approach has recently been proposed [61]. To this end, Mukundan presented a series of moments in [62] that uses discrete Tchebichef polynomials to analyze the image.

Typically, the extraction approaches are divided into global and local features. The former is also called a holistic-based approach [63], which can capture the essential characteristics of the full human face image. At the same time, it is known as the component-based approach or block-processing-based approach, from specific areas in images [64]. In the global feature-based approach, various imaging setups are used to achieve improved performance for feature extraction [65]. To this end, several feature extraction techniques have been proposed so far to enable a global feature-based approach [66,67]. In block processing or what is known as the local feature-based approach, the image features can be extracted locally by utilizing OMs, which entails processing the image’s blocks after it has been divided into several blocks to ease their processing. In this approach, the signals such as images and videos can be divided efficiently into several blocks so that they transfer to another domain to extract the features [68]. The signal characteristics can be stored locally in memory to prepare it for the next step of processing. The work in [63] demonstrates that the (local) block-processing-based approach achieves better performance in feature extraction compared with the (global) holistic-based approach.

One technique for extracting local features is the local binary patterns method [69,70,71]. In addition, the combination of global- and local-based approaches, which is termed the hybrid features extraction-based approach, aims to achieve the highest object recognition accuracy [72,73]. It is demonstrated that block processing, which represents local feature extraction, can achieve the highest recognition accuracy with the trade-off of higher computation complexity. Specifically, compared with global features, local features are thought to be more reliable and improve recognition accuracy, see, e.g., [74,75,76]. To this end, partitioning the images using image block processing has the potential of extracting the blocks of any image and analyzing them sequentially. From the perspective of computer memory, this operation is not sequential, which is seen as a major flaw in performance and a crucial difference between the memory and the speed of the CPU. While such an operation would result in additional cache misses and replacements, accessing the complete matrix sequentially can aid in maintaining spatial locality [68]. The removal of additional procedures will speed up the extraction of local features. Specifically, extracting local features from the image blocks using discrete transform will decrease the computational complexity, which is called a fast overlapped block-processing method for feature extraction [68]. Although several advanced methods have been proposed for object recognition, the accuracy and running time are to date considered challenging issues that need to be addressed. Therefore, finding a quick and accurate mechanism for 3D object detection is necessary. Additionally, most of the exciting works need to account for the impact of undesirable noise on recognition. Hence, there is a limited understanding of the effect of noisy environments. Therefore, investigating the proposed method in the noise condition is significant to characterize the effectiveness of the feature extraction for object recognition processes.

### 1.2. Paper Contributions

To overcome the aforementioned challenges, a robust object recognition algorithm that exploits Charlier polynomials and their moments is proposed. The proposed algorithm has a powerful capability for the characterization and feature extraction of the signals of the 3D objects effectively. In addition, to extract the features effectively and in a fast manner, this paper exploits an overlap block-processing technique to provide a construction of auxiliary matrices, which essentially extends the original signal to prevent the time delay in the loops computation. Furthermore, the proposed method is evaluated in the noise condition to characterize the effectiveness of the proposed method in feature extraction for object recognition processes. The major contributions of this paper can be summarized as follows: (1) Proposing an advanced design for robust 3D object recognition, which takes into account the accuracy, computational complexity, and execution time. (2) Exploiting the powerful Charlier polynomials to extract the features of the 3D objects. (3) Developing a fast overlapped block-processing algorithm, which shows more accurate processing for the blocks of the image to perform fast feature extraction with low complexity. The proposed overlapped block-processing method is mainly used to decrease the computation time. (4) Finally, implementing the support vector machine (SVM) to classify object recognition features accurately. To this end, a well-known dataset known as the McGill benchmark dataset is used for performance evaluation [77]. The results demonstrate that the proposed method achieves high recognition accuracy with lower computational complexity. Furthermore, the results demonstrate that the proposed method is able to reduce noise distortion and outperforms traditional methods under both clean and noisier environments. These achievements signify the importance of the proposed method for the future implementation of 3D object recognition.

### 1.3. Paper Organization

The paper is organized as follows. In Section 2, the orthogonal polynomials and their moments are introduced. In Section 3, the methodology of the proposed method for feature extraction and recognition of 3D objects is presented. In Section 4, the performance evaluation of the proposed method and the numerical results are discussed. Finally, the conclusion of the paper is presented.

## 2. Preliminaries of OPs and Their OMs

The mathematical definition of the utilized OPs is explained in this section. Additionally, this section also describes the computation of the OMs for the 3D signals.

### 2.1. Charlier Polynomials Computation and Their Moments

This subsection discusses the Charlier polynomials and their moments. In addition, the existing three-term recurrence (TTR) relation is described. Several studies have considered the use of Charlier polynomials due to its accuracy and effectiveness [78]. To this end, research on the application of Charlier polynomials has been divided into two main areas: moment computation algorithms and recurrence relation algorithms. For the recurrence relation-based algorithms, the research works exploit the n-direction and x-direction of the matrix. However, generating high-order polynomials is not possible in these recurrence algorithms. This is due to the use of the initial values and the number of recurrence times. The research works make use of either the *x*-direction or the *n*-direction of the recurrence algorithm as their calculation algorithms. To the best of our knowledge, no research studies have looked into using Charlier polynomials and their moments for 3D object detection. This paper investigates the effect of using Charlier polynomials for 3D object recognition. This paper also aims to provide an efficient method for achieving a recurrence relation to compute Charlier polynomials for high-order polynomials.

In what follows, the Charlier polynomials and their moments computation are presented.

### 2.2. Computation of Charlier Polynomials

Charlier polynomials $C_{n}\left(y;p\right)$ of *d*th dimension can be calculated as follows:

(1) $$\begin{array}{c} C_{n}\left(x;p\right)={}_{2}F_{0}\left(\begin{array}{c} -n,-x\\ \_ \end{array}|\;-\frac{1}{p}\right),\\ n,x=0,1,2\cdots ,N-2,N-1;\;p>0, \end{array}$$

where *p* denotes the parameter of the Charlier polynomials, and ${}_{2}F_{0}$ represents the mathematical formulation of the hypergeometric series, which is expressed as [79]

(2) $${}_{2}F_{0}\left(\begin{array}{c} a_{1},a_{2}\\ \;\;\_ \end{array}|\;z\right)=\sum_{k=0}^{\infty }\frac{{\left(a_{1}\right)}_{k}\;{\left(a_{2}\right)}_{k}}{k!}{\left(z\right)}^{k},$$

where ${\left(a\right)}_{k}$ denotes the ascending factorial, which is termed as the Pochhammer symbol [79].

Following the expressions provided by Equations (1) and (2), Charlier polynomials can be written as

(3) $$C_{n}\left(x;p\right)=\sum_{k=0}^{\infty }\frac{{\left(-n\right)}_{k}\;{\left(-x\right)}_{k}\;}{k!}{\left(-\frac{1}{p}\right)}^{k}.$$

It is worth noting that the orthogonality condition should be met with Charlier polynomials. Besides, the weighted function can be applied to the Charlier polynomials so that

(4) $$\sum_{x=0}^{D}C_{n}\left(x;p\right)\;\;C_{m}\left(x;p\right)\;\;\omega_{C}\left(x;p\right)=\rho_{C}\left(n;p\right)\;\delta_{nm},$$

where D=N−1, $\omega_{C}\left(x;p\right)$, which denotes the weighted function and $\rho_{C}\left(d;p\right)$ represents the squared norm of Charlier polynomials dx. The weighted function and the squared norm of Charlier polynomials are provided in expressions (5) and (6), respectively.

(5) $$\omega_{C}\left(x;p\right)=\frac{e^{-p}p^{x}}{x!}.$$

(6) $$\rho_{C}\left(n;p\right)=\frac{n!}{p^{n}}.$$

It is worth noting that the calculation of the Charlier polynomials’ coefficients provided by the expression in Equation (3) may cause numerical instability. Hence, to overcome this issue, a weighted normalized Charlier polynomial is applied. To this end, the *n*th order of weighted normalized Charlier polynomials can be expressed as

(7) $${\hat{C}}_{n}\left(x;p\right)=\sqrt{\frac{\omega_{C}\left(x;p\right)}{\rho_{C}\left(n;p\right)}}\;C_{n}\left(x;p\right).$$

### 2.3. Computation of Charlier Moments

This subsection discusses the computation of Charlier moments. The Charlier moments, denoted as transform coefficients, are scalar quantities utilized to demonstrate signals without redundancy [49,80]. For a one-dimensional (1D) signal, denoted as f(x), Charlier moments can be computed in the moment domain as

(8) $$\begin{array}{c} \mu_{n}=\sum_{x=0}^{N-1}{\hat{C}}_{n}\left(x;p\right)\;f\left(x\right)\\ n=0,1,\cdots ,Ord-1, \end{array}$$

where $\mu_{n}$ denotes the Charlier moments and Ord represents the maximum number of orders utilized for signal representation. To obtain the signal $\bar{f}\left(x\right)$ from the Charlier domain (moments domain), inverse transform can be utilized as follows:

(9) $$\bar{f}\left(x\right)=\sum_{n=0}^{Ord-1}\mu_{n}\;{\hat{C}}_{n}\left(x;p\right)$$

For a two-dimensional (2D) signal f(x,y) of size N×M, the Charlier moments with 2D signal, denoted as $\mu_{nm}$, can be computed as

(10) $$\begin{array}{ccc} & & \mu_{nm}=\sum_{x=0}^{N-1}\sum_{y=0}^{M-1}f\left(x,y\right){\hat{C}}_{n}\left(x;p\right)\;{\hat{C}}_{m}\left(y;p\right)\\ & & n=0,1,\cdots ,Ord_{1}\;\mathrm{and}\;\\ & & m=0,1,\cdots ,Ord_{2}, \end{array}$$

where the parameters $Ord_{1}\;\mathrm{and}\;Ord_{2}$ denote the highest order used for the representation of the signal. To reconstruct the 2D signal $\bar{f}\left(x,y\right)$, denoted as $\bar{f}=f$, from the Charlier domain, the following inverse transformation is used:

(11) $$\bar{f}\left(x,y\right)=\sum_{n=0}^{Ord_{1}}\sum_{m=0}^{Ord_{2}}\mu_{nm}\;{\hat{C}}_{n}\left(x;p\right)\;{\hat{C}}_{m}\left(y;p\right)$$

To compute the moments for higher dimensional space, in our case, the 3D signal, f(x,y,z), the following formula is used:

(12) $$\begin{array}{ccc} & & \mu_{nml}=\sum_{x=0}^{N-1}\sum_{y=0}^{M-1}\sum_{z=0}^{L-1}f\left(x,y,z\right)\;{\hat{C}}_{n}\left(x;p\right)\;{\hat{C}}_{m}\left(y;p\right)\;{\hat{C}}_{l}\left(z;p\right)\\ & & n=0,1,\cdots ,Ord_{1},\;\\ & & m=0,1,\cdots ,Ord_{2}\;\mathrm{and}\;\\ & & l=0,1,\cdots ,Ord_{3}. \end{array}$$

### 2.4. Charlier Coefficients Computation Using Recurrence Relation Algorithm

This section presents the algorithm exploited to compute the coefficients of Charlier polynomials. It is worth noting that the algorithm used in this paper is the recurrence relation, which has been presented in [78].

The computation of initial values of Charlier polynomials’ coefficients is essential for obtaining an efficient and reliable recurrence relation algorithm. It should be noted that both three-term recurrence relations algorithms in the *x*-direction and the *n*-direction depend on two sets of initial values. To this end, ${\hat{C}}_{0}\left(x;p\right)$ and ${\hat{C}}_{1}\left(x;p\right)$ are the two initial values used in the three-term recurrence relation algorithm in the *x*-direction. In general, calculating the set of initial values is mathematically intractable. This is attributed to incorrectly computed values. To address this issue, a logarithmic function is used [78]:

(13) $${\hat{C}}_{p}\left(0;p\right)={\left(e^{-\left(log\Gamma (p)+p+(1-p)log(p)\right)}\right)}^{0.5},$$

where logΓ denotes the logarithmic mathematical operation for the gamma function.

To obtain the remaining Charlier polynomials’ coefficients for ${\hat{C}}_{n}\left(0;p\right)$, the two-term recurrence relation is utilized. We have two ranges for x=1, which are n<p−1 and n>p. For the range n<p−1, n=p−2,p−3,⋯,0, the two-term recurrence relation can be written as

(14) $$\begin{array}{ccc} {\hat{C}}_{n-1}\left(0;p\right) & = & \sqrt{\frac{n}{p}}\;\;{\hat{C}}_{n}\left(0;p\right). \end{array}$$

For the range n>p, n=p+1,p+2,⋯,N−1, the following expression is used:

(15) $$\begin{array}{ccc} {\hat{C}}_{n}\left(0;p\right) & = & \sqrt{\frac{p}{n}}\;{\hat{C}}_{n-1}\left(0;p\right) \end{array}$$

After computing the coefficients for x=0, they are used to compute the Charlier polynomials’ coefficients for x=1 using the following recurrence relation:

(16) $$\begin{array}{ccc} {\hat{C}}_{n}\left(1;p\right) & = & \frac{\left(p-n\right)}{\sqrt{n}}\;{\hat{C}}_{n}\left(0;p\right)\\ n & = & 0,1,\cdots ,N-1. \end{array}$$

**Figure 1:**
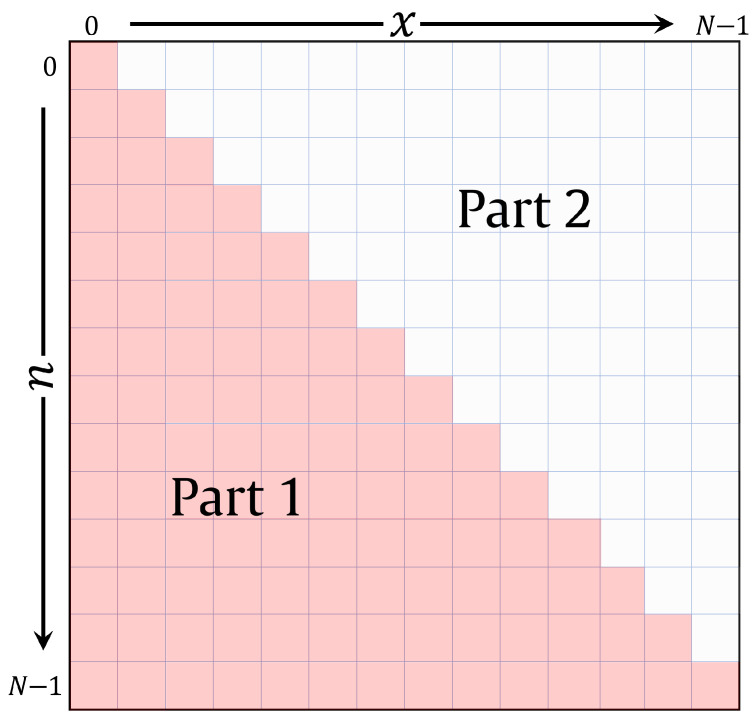
An illustration of the plan dividing into n−x parts for Charlier polynomials.

To this end, the polynomial space of the Charlier polynomials is divided into two portions: lower triangle and upper triangle [78]. These portions are known as “Part 1” and “Part 2”, which are shown in Figure 1. Charlier polynomials’ coefficients in the lower triangle matrix (“Part 1”) are obtained using three three-term recurrence relations. In addition, Charlier polynomials’ coefficients in the upper triangle matrix (“Part 2”) are obtained using the symmetry relation provided in the expression given by

(17) $$\begin{array}{ccc} & & {\hat{C}}_{n}\left(x;p\right)={\hat{C}}_{x}\left(n;p\right)\\ & & n=0,1,\cdots ,N-1,\;\mathrm{and}\;\\ & & x=0,1,\cdots ,n-1. \end{array}$$

After the weighted normalized Charlier polynomials’ identity and initial values calculations are presented, the calculation of the Charlier polynomials’ coefficients in “Part 1” is performed by exploiting the three-term recurrence in the *x*-direction. To this end, the following calculations are

(18) $${\hat{C}}_{n}\left(x+1;p\right)=A\;{\hat{C}}_{n}\left(x;p\right)+B\;{\hat{C}}_{n}\left(x-1;p\right),$$

where x=1,2,⋯,N−1andn=x,x+1,⋯,N−1; the parameters *A* and *B* are obtained, respectively, as [78]

(19) $$\begin{array}{ccc} A & = & \frac{a-n+x}{\sqrt{a(x+1)}}, \end{array}$$

(20) $$\begin{array}{ccc} B & = & -\sqrt{\frac{x}{\left(x+1\right)}}. \end{array}$$

For more clarification, the utilized algorithm for the weighted normalized Charlier polynomials is summarized in Algorithm 1.
**Algorithm 1** The algorithm used to compute the Charlier polynomials**Input:***N* = Polynomial size, *p* = Polynomial parameter.**Output:**$\hat{C}$ = Charlier polynomials.1: Initialize $\hat{C}$ with a size of N×N2: ${\hat{C}}_{p}\left(0;p\right)\leftarrow {\left(e^{-\left(log\Gamma (p)+p+(1-p)log(p)\right)}\right)}^{0.5}$ {Compute initial value}.3: n←[p−2,p−1,⋯,0] {Set the range of *n*}.4:**for** *i* in range *n* **do**5:   ${\hat{C}}_{i-1}\left(0;p\right)\leftarrow \sqrt{\frac{i}{p}}\;\;{\hat{C}}_{i}\left(0;p\right)$6: **end for**7: n←[n=p+1,p+2,⋯,N−1,] {Set the range of *n*}.8: **for** *i* in range *n* **do**9:   ${\hat{C}}_{i}\left(0;p\right)\leftarrow \sqrt{\frac{p}{n}}\;{\hat{C}}_{i-1}\left(0;p\right)$10:**end for**11:**for** n=0 to N−1 **do**12:   ${\hat{C}}_{n}\left(1;p\right)\leftarrow \frac{\left(p-n\right)}{\sqrt{n}}\;{\hat{C}}_{n}\left(0;p\right)$13:**end for**14:{Compute the coefficients in “Part 1”}15:**for** x=1 to N−1 **do**16:    **for** n=x to N−1 **do**17:      $A\leftarrow \frac{a-n+x}{\sqrt{a(x+1)}}$18:      $B\leftarrow -\sqrt{\frac{x}{\left(x+1\right)}}$19:      ${\hat{C}}_{n}\left(x+1;p\right)\leftarrow A\;{\hat{C}}_{n}\left(x;p\right)+B\;{\hat{C}}_{n}\left(x-1;p\right)$20:    **end for**21: **end for**22: {Compute the coefficients in “Part 2”}23:**for** n=1 to N−1 **do**24:    **for** x=0 to n−1 **do**25:       ${\hat{C}}_{n}\left(x;p\right)\leftarrow {\hat{C}}_{x}\left(n;p\right)$26:    **end for**27: **end for**28: **return** {$\hat{C}$ Note that $H_{d}$ in Equation (27) is equal to $\hat{C}$.}

## 3. Methodology of the Proposed Feature Extraction and Recognition Method of 3D Object

This section presents the feature extraction and recognition processes for the presented 3D object recognition algorithm.

**Figure 2:**
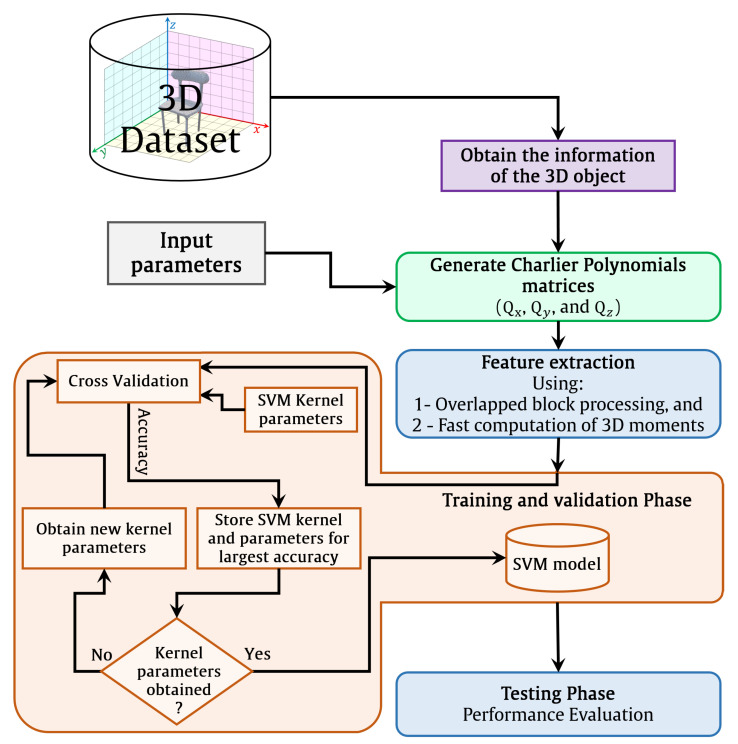
The brief methodology of the presented 3D recognition algorithm.

For any recognition system, a feature extraction process is employed to represent signals. As a result, local feature extraction can be used to enable more effective object recognition systems rather than global feature extraction due to their effectiveness, as discussed earlier in the introduction. Therefore, the 3D image might be separated into blocks to increase recognition accuracy. Each block has a size of $B_{x}\times B_{y}\times B_{z}$. The Charlier polynomials are generated using the procedures in Section 2.4, where Charlier polynomials can be generated with parameter *p*. The brief methodology of the presented 3D recognition algorithm is shown in Figure 2. First, the 3D image information is obtained. Then, the Charlier polynomials are generated with parameter *p*. Next, the overlapped polynomials are generated to reduce the computation cost. After that, the fast 3D moments’ computation is used to transform the 3D images into the moment domain. Finally, the features are normalized and used to train the SVM model for recognition.

**Figure 3:**
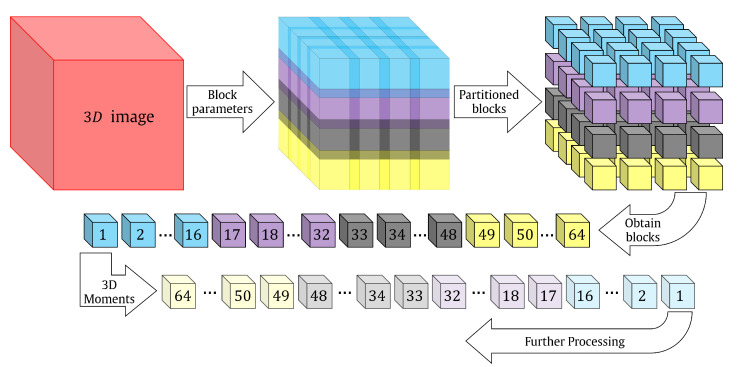
Flow diagram of the traditional block-processing algorithms.

The global-based feature extraction approach is, to some extent, inaccurate for noisy environments, which highly impedes the characterization of efficient 3D object algorithms in more realistic settings. Moreover, the performance of 3D object recognition accuracy may be degraded in noisy environments [45,81]. Therefore, preprocessing for the 3D object becomes essential to mitigate the effect of noise but it may come at the expense of increasing the computation complexity. The extraction of local features leads to a high computation cost because the traditional method is used, which is considered a bottleneck for real-time application [82]. The local features are extracted after partitioning the 3D object into sub-blocks. For more clarification, see Figure 3.

**Figure 4:**
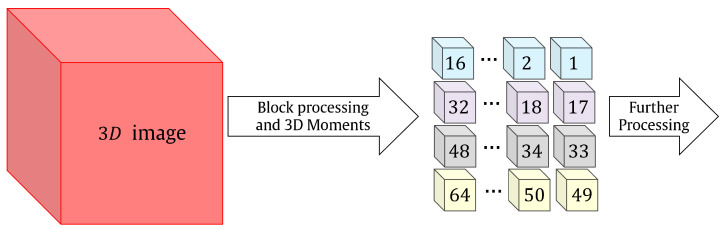
Flow diagram of the proposed fast block-processing algorithm.

A fast overlapped block-processing technique is exploited to overcome the above challenges. To extract local features, most applications use a non-overlapping block-processing technique. On the other hand, overlapped block processing could enhance the accuracy of 3D object recognition [45,81]. Typically, the processing of the blocks in parallel will significantly raise the cost of computing. We solved this issue by using the fast overlapped block processing described in [68]. The fundamental idea behind fast overlapped block processing (FOBP) is to extend the image by adding auxiliary matrices, which does away with the requirement for nested loops. The computing cost of the feature extraction procedure will be drastically reduced by eliminating the nested loops (see Figure 4). Suppose a 3D image F with a size of $N_{x}\times N_{y}\times N_{z}$ needs to be partitioned into overlapped blocks. The size of the blocks are $B_{x}\times B_{y}\times B_{z}$ with overlapping sizes of $v_{x}$, $v_{y}$, and $v_{z}$ in the *x*, *y*, and *z*-direction, respectively. This lead to a total blocks ($T_{Blocks}$) of

(21) $$\begin{array}{ccc} T_{Blocks} & = & Blocks_{x}\times Blocks_{y}\times Blocks_{z},\\ T_{Blocks} & = & \frac{N_{x}}{B_{x}-2v_{x}}\times \frac{N_{y}}{B_{y}-2v_{y}}\times \frac{N_{z}}{B_{z}-2v_{z}}. \end{array}$$

**Figure 5:**
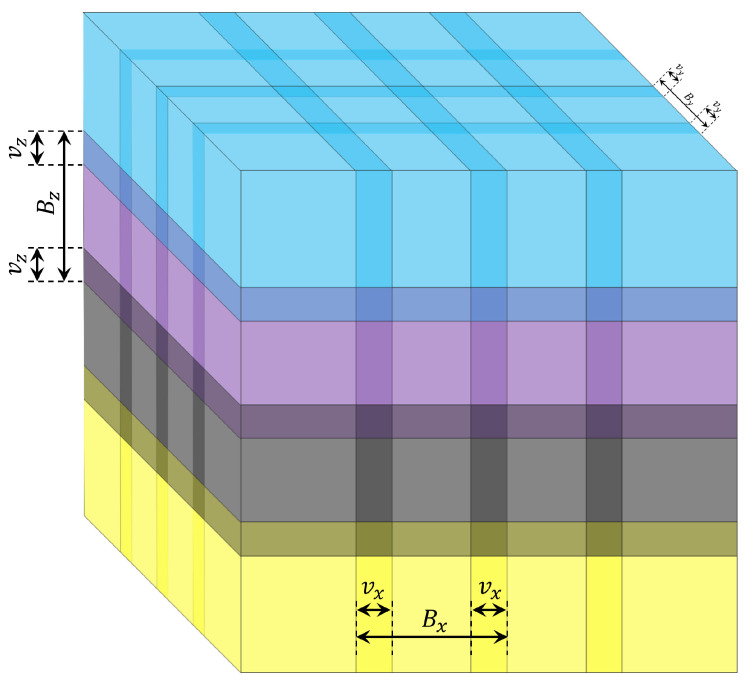
Details of the 3D image with overlapped blocks.

For further details about the expressions above, see Figure 5.

**Figure 6:**
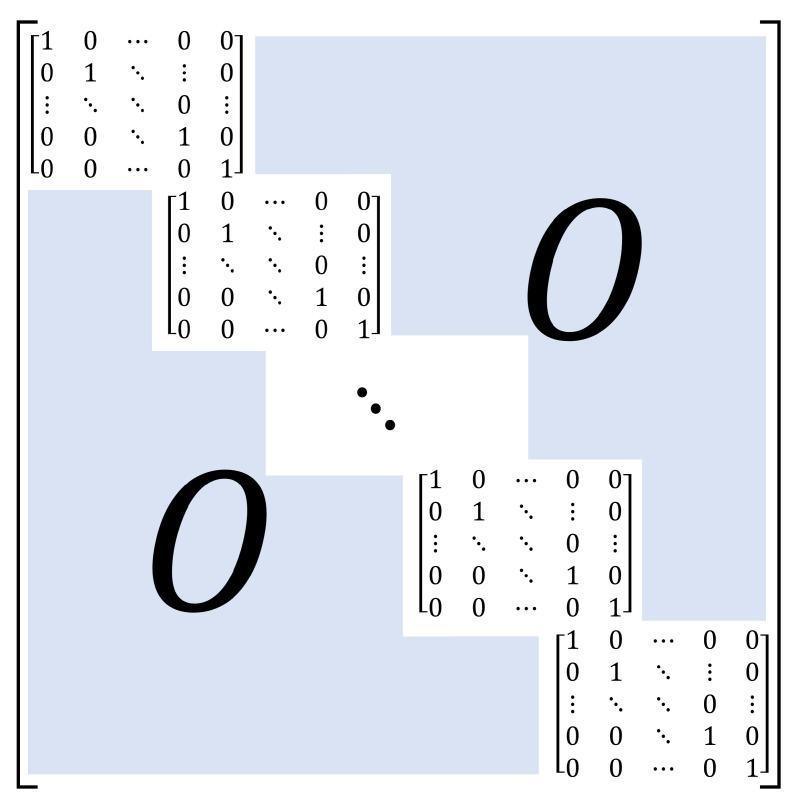
Details of $E_{d}$ matrix. The size of $E_{d}$ matrix is $B_{d}\cdot Blocks_{d}\times N_{d}$. The size of the identity matrix is $B_{d}\times B_{d}$.

Suppose the matrix G represents the extended version of F and can be computed as follows [68]:

(22) $$G=\sum_{z}E_{z}\left[E_{y}\;F\;E_{x}^{T}\right],$$

where $E_{x}$, $E_{y}$, and $E_{z}$ are rectangular matrices with sizes of $\left(B_{x}\cdot Blocks_{x}\times N_{x}\right)$, $\left(B_{y}\cdot Blocks_{y}\times N_{y}\right)$, and $\left(B_{z}\cdot Blocks_{z}\times N_{z}\right)$, respectively. For further elucidation, the matrix $E_{d}$ is shown in Figure 6, where *d* represents the dimensions (*x*, *y*, and *z*).

To compute the moments (M) for a 3D image using matrix multiplication, Equation (12) can be rewritten as follows [83]:

(23) $$\begin{array}{cc} M & =\sum_{z}R_{z}\left[R_{y}\;G\;R_{x}^{T}\right] \end{array}$$

By substituting Equation (22) in Equation (23), we obtain

(24) $$\begin{array}{cc} M & =\sum_{z}R_{z}\left[R_{y}\;\left[\sum_{z}E_{z}\left[E_{y}\;F\;E_{x}^{T}\right]\right]\;R_{x}^{T}\right]. \end{array}$$

Note that M represents the matrix form of the moments $\mu_{nml}$.

**Figure 7:**
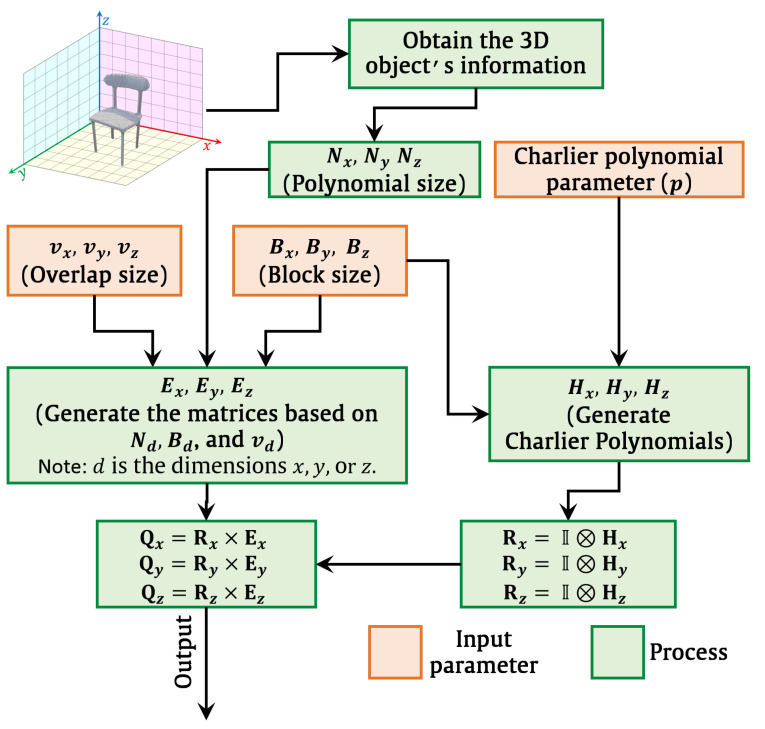
Flow diagram demonstrates the generation of the matrices $Q_{x}$, $Q_{y}$, and $Q_{z}$.

By following the proof presented in [68], Equation (24) can be rewritten as follows:

(25) $$M=\sum_{z}Q_{z}\left[Q_{y}GQ_{x}^{T}\right],$$

where $Q_{d}$ are computed as follows:

(26) $$Q_{d}=R_{d}\;E_{d},$$

where the matrix $R_{d}$ can be obtained as follows:

(27) $$R_{d}=I⊗H_{d},$$

where matrix I denotes an identity matrix, ⊗ denotes the Kronecker product, and $H_{d}$ represents the Charlier polynomials. Note that *d* represents the dimensions *x*, *y*, or *z*. Due to the matrices independence from the image, they are generated, stored, and repeatedly used [45,68]. The process for the matrices generation is depicted in Figure 7.

After the matrices ($Q_{x}$, $Q_{y}$, and $Q_{z}$) are generated, the images are transformed into the Charlier moment domain to extract features (see Algorithm 2). Then, these features are normalized to obtain the feature vector. Finally, the objects are classified based on the extracted features.
**Algorithm 2** The 3D moments computation [83]**Input:**F = 3D image, $Q_{d}$ = Charlier polynomials.**Output:**FV = Charlier moments.1: Generate extended 3D image (G) from the 3D image F {Equation (22).}2: Get stored Charlier polynomials $Q_{x}$, $Q_{y}$, and $Q_{z}$ {Using Equation (26).}3: **for** *z* = 1 to $Ord_{z}$ **do**4:     $M\leftarrow M+R_{z}⊗\left[Q_{x}\;G\;Q_{y}\right]$5: **end for**6: FV←reshape(M) {Reshape the computed moments as a feature vector.}7: **return** FV {Note: in the training and testing phases, the feature vector is normalized.}

In this paper, the normalized feature vector is obtained and considered an input to the classifier. To this end, a label (ID) is considered for each input image of the objects. The classification procedure is performed in this paper using SVM. The SVM technique is selected here due to its effectiveness in optimizing the margin between hyperplane separation classes and data [84]. Furthermore, the SVM technique can be very efficient for object recognition. This is attributed to the fact that SVM is more robust to signal fluctuation [85]. In this paper, LIB-SVM is used in the classification process [86].

**Figure 8:**
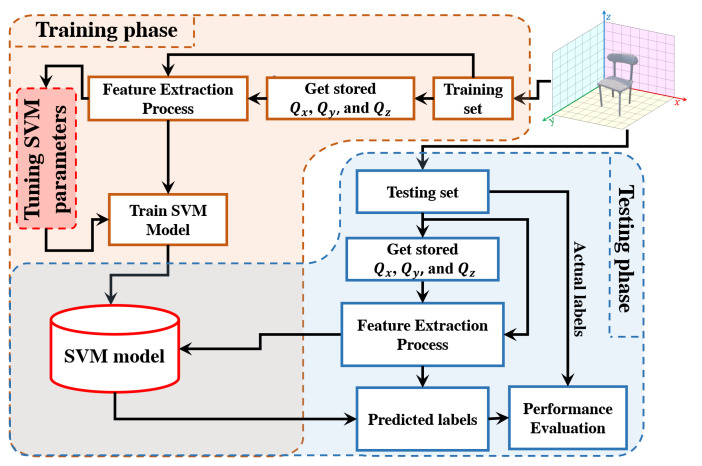
A diagram shows the flow process for the proposed 3D object recognition method.

Figure 8 shows a model of the proposed 3D object recognition method.

## 4. Experiments and Discussions

**Figure 9:**
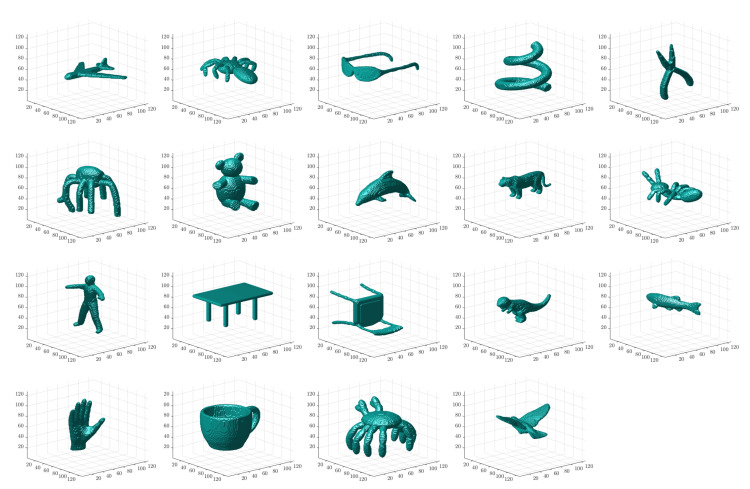
An illustration of samples of 3D objects.

In this section, the performance of the proposed Charlier polynomials algorithm for 3D object recognition is evaluated. In this experiment, the well-known McGill dataset—developed in [77]—is used as a benchmark dataset. In particular, this dataset contains 19 classes, denoted as 3D objects. These 3D objects are named as planes, spiders, spectacles, snakes, pliers, octopus, teddies, dolphins, fours, ants, humans, tables, chairs, dinosaurs, fishes, hands, cups, craps, and birds. Samples of the aforementioned 3D objects are shown in Figure 9.

In this experiment, the results are obtained over 19 different objects and various effects. These effects are translations and rotations. The sample objects are translated in the *x*, *y*, and *z* axes and their combinations (xy, xz, yz, and xyz) range from (1, 1, 1) to (10, 10, 10) with a step of (1, 1, 1). In addition, for each direction, the sample objects are rotated in the *x*, *y*, *z*, xy, xz, yz, and xyz axes between 10${}^{\circ }$ and 360${}^{\circ }$ with a step of 10${}^{\circ }$. The resulting number of samples per object is 1252, which produces a total number of 23,788 samples for all objects.

The flow diagram process of the 3D object recognition is shown in Figure 8. For the 3D object recognition, different block sizes are considered in this experiment, which are given by the block sizes of 64×64×64, 32×32×32, and 16×16×16. Table 1, Table 2 and Table 3 present the performance results of block sizes of 64×64×64, 32×32×32, and 16×16×16, respectively. Besides, different overlap sizes are also considered in addition to the sizes of the testing and training sets considered during this experiment, which are given as 70% and 30%, respectively. As discussed earlier, the proposed solution for 3D object recognition and feature extraction is Charlier polynomials (see Algorithm 1) with parameter p=BlockSize/2. The SVM model is used in the proposed algorithm for object classification. The LIB-SVM library developed in [86] is used to train the extracted features. The SVM kernel exploits LIB-SVM and uses the radial basis function. In the training phase, five-fold cross-validation is utilized to obtain the values of the SVM parameters (see Figure 2). The recognition accuracy is the number of correct predictions divided by the total number of predictions as follows:

(28) $$Accuracy=\frac{Number\;\;of\;\;correct\;\;predictions}{Total\;\;number\;\;of\;\;predictions}$$

Table 1, Table 2 and Table 3 reported the recognition rate for clean and noisy environments. First, we will discuss the clean environment results; then, the noisy environment will be considered successively for different types of noise.

The results in Table 1 show that the accuracy of block size of 64×64×64 starts at 68.25% and increases to 80.04% as the overlap block size is increased from 0×0×0 to 16×16×16, which shows an improvement ratio of 14.73%. This implies that increasing the overlap block size can help in improving the recognition accuracy. For the block size of 32×32×32 given in Table 2, the object recognition accuracy starts with a value of 76.28% at overlap size of 0×0×0, which achieves an accuracy improvement of 8.03% higher than that obtained with the block size of 64×64×64 at an overlap size of 0×0×0. In addition, the object recognition accuracy of the block size of 32×32×32 is increased to 80.10% at an overlap block size of 4×4×4. For the block size of 16×16×16 given in Table 3, the object recognition accuracy is increased from 70.58% to 80.10% as the overlap size is increased from 0×0×0 to 2×2×2.

To this end, the highest accuracy performance is achieved at a block size of 64×64×64 when an overlap size of 16×16×16 is used, at a block size of 32×32×32 when an overlap size of 4×4×4 is exploited, and at a block size of 16×16×16 when an overlap size of 2×2×2 is utilized, as illustrated in Table 1, Table 2 and Table 3, respectively. In a nutshell, the best accuracy can be achieved when the overlap block size is increased and the block size is decreased.

Different noisy environments are considered for further clarification and evaluation of the proposed object recognition method, and the results for each type are reported. It is worth noting that (GN) stands for Gaussian noise, (SPN) stands for salt-and-pepper noise, and SPKN stands for Speckle noise. Note that different noise levels are considered for each type of noise. From Table 1, it is obvious for the case of GN with all its different densities values from 0.0001 to 0.0005 that the accuracy is increased as the values of the overlap block size are increased. In addition, the same observation is perceived for SPN and SPKN for all noise density values. Moreover, Table 2 shows that for all types of noise and noise densities, higher accuracy is achieved at the highest overlap block size. On the other hand, for a block size of 16×16×16, higher accuracy is obtained for the overlap block size equal to 1×1×1 for all noisy environments.

The results show that the best-case scenario can be obtained at a block size of 32×32×32 and an overlap size of 4×4×4. The accuracy of object recognition starts at very low values with the block size of 64×64×64, which is given in Table 1 when an overlap size of 0×0×0 is considered. The recognition accuracy is then increased to the highest values at a block size of 64×64×64 and an overlap size of 16×16×16, as given in Table 1; a block size of 32×32×32 and overlap size of 4×4×4, as shown in Table 2; and a block size of 16×16×16 and overlap size of 2×2×2, as demonstrated in Table 3.

To evaluate the performance of the presented algorithm, a comparison is performed with existing works in terms of recognition accuracy. The existing works in the comparison are Geometric Moment Invariants (GMI), Tchebichef–Tchebichef–Krawtchouk Moment Invariants (TTKMI), Tchebichef–Krawtchouk–Krawtchouk Moment Invariants (TKKMI), Krawtchouk–KrawtchoukKrawtchouk Moment Invariants (KKKMI), TchebichefTchebichef–Tchebichef Moment Invariants (TTTMI), Hahn Moment Invariants (HMI), Krawtchouk Moment Invariants (KMI), Tchebichef Moment Invariants (TMI), and Direct Krawtchouk Moment Invariants (DKMI). The results of the presented and existing works are reported in Table 4.

It can be observed from Table 4 that the average recognition accuracy of the presented algorithm outperforms the accuracy of the existing works. According to the results obtained from this table, the recognition accuracy of the presented algorithm is significantly high compared with that computed from the existing algorithms for all the given values of the block size (64×64×64, 32×32×32, and 16×16×16) and overlap size (16×16×16, 4×4×4, and 1×1×1). Therefore, it can be concluded that the presented algorithm can be useful in object recognition applications.

**Figure 10:**
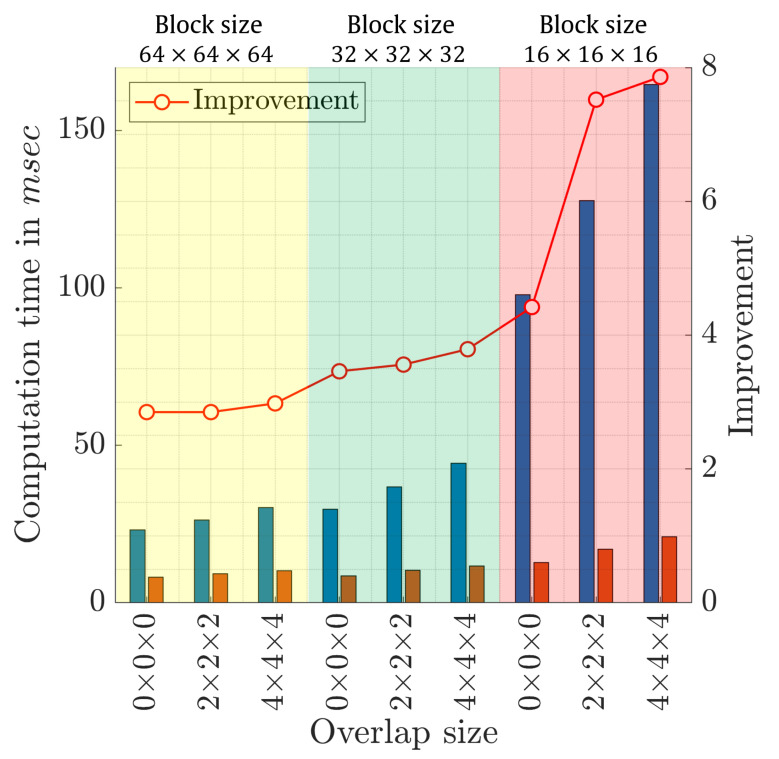
Computation time in msec comparing the proposed and traditional algorithms.

Furthermore, in order to provide further performance evaluation of the proposed method, the computation time of the proposed algorithm is compared with the traditional algorithm. To this end, Figure 10 illustrates an average computation time for 10 runs for both the proposed and traditional algorithm under different values of block sizes and overlap sizes. In addition, the percentage performance improvement between the proposed algorithm and the traditional algorithm is also provided. This percentage performance improvement is obtained by dividing the results from the traditional algorithm by those obtained from the proposed algorithm. Figure 10 shows that the proposed algorithm significantly outperforms the traditional algorithm, where the average performance improvement across whole values is recorded as around 4.70 compared with the traditional algorithm. The proposed recognition algorithm achieves the highest percentage performance improvement when the block size is 16×16×16 with an overlapped size of 4×4×4, which is recorded as 7.86. This clearly signifies the robustness of our algorithm when a small block size, i.e., 16×16×16, is considered.

## 5. Conclusions

This paper presents an efficient algorithm for 3D object recognition with low computational complexity and fast execution time based on Charlier polynomials. The proposed algorithm has a powerful capability for extracting the features of the 3D object in a fast manner. This was attributed to the overlapped block-processing technique, which allows the signals to be virtually extended to auxiliary matrices to avoid the time delay during loops computation. In addition, in order to characterize the effectiveness of the proposed 3D object recognition method, a noise environment was considered in the evaluation and comparison. This paper also implemented the SVM algorithm to classify the 3D object features. The proposed 3D object recognition method was evaluated under different environments. The results illustrate that the proposed 3D object approach achieved high recognition accuracy as well as low computation time under the different noisy environments considered. This achievement signifies the importance of the proposed 3D object recognition method for future applications.

## Figures and Tables

**Table 1:** Evaluation of the accuracy (%) of the proposed algorithm of 3D object recognition for the block size of (64×64×64) under different environments.

	Block Size = 64×64×64				
	**Overlap Size (** $v_{x}\times v_{y}\times v_{z}$ **)**				
**Environment**	**0**	**2**	**4**	**8**	**16**
Clean	68.25	70.42	72.44	78.52	80.04
GN 0.0001	39.44	61.18	68.96	78.58	80.16
GN 0.0002	36.95	57.48	67.84	78.58	80.18
GN 0.0003	35.32	55.44	67.14	78.59	80.13
GN 0.0004	33.75	53.44	66.67	78.65	80.01
GN 0.0005	32.26	51.38	66.23	78.63	80.03
SPN 0.1	37.86	58.80	68.36	78.56	80.20
SPN 0.2	31.33	49.28	65.53	78.62	80.04
SPN 0.3	29.39	42.71	61.52	78.36	79.72
SPN 0.4	27.15	39.24	57.66	78.14	78.97
SPN 0.5	26.09	36.56	52.81	77.67	78.32
SPKN 0.2	68.24	70.41	72.40	78.55	80.03
SPKN 0.4	68.22	70.47	72.33	78.53	80.04
SPKN 0.6	68.17	70.39	72.37	78.46	80.04
SPKN 0.8	68.25	70.35	72.43	78.48	79.97
SPKN 1.0	68.21	70.51	72.30	78.53	80.03

**Table 2:** Evaluating the accuracy (%) of the proposed algorithm of 3D object recognition for the block size of (32×32×32) under different environments.

	Block Size = 32×32×32			
	**Overlap Size (** $v_{x}\times v_{y}\times v_{z}$ **)**			
**Environment**	**0**	**1**	**2**	**4**
Clean	76.28	77.18	78.86	80.10
GN 0.0001	76.21	77.15	78.79	80.16
GN 0.0002	76.16	77.11	78.73	80.14
GN 0.0003	76.18	77.02	78.67	80.11
GN 0.0004	75.98	77.01	78.60	80.14
GN 0.0005	75.75	76.98	78.62	80.14
SPN 0.1	76.25	77.21	78.80	80.16
SPN 0.2	75.46	76.94	78.56	80.10
SPN 0.3	74.39	76.56	78.22	79.92
SPN 0.4	73.79	75.64	78.00	79.58
SPN 0.5	73.03	74.78	77.64	79.21
SPKN 0.2	76.25	77.22	78.90	80.11
SPKN 0.4	76.26	77.18	78.87	80.13
SPKN 0.6	76.33	77.17	78.90	80.11
SPKN 0.8	76.28	77.21	78.84	80.13
SPKN 1.0	76.25	77.18	78.84	80.04

**Table 3:** Evaluation of the accuracy (%) of the proposed algorithm of 3D object recognition for the block size of (16×16×16) under different environments.

	Block Size = 16×16×16		
	**Overlap Size (** $v_{x}\times v_{y}\times v_{z}$ **)**		
**Environment**	**0**	**1**	**2**
Clean	70.58	80.24	80.10
GN 0.0001	70.73	80.31	80.08
GN 0.0002	70.75	80.27	80.08
GN 0.0003	70.76	80.30	80.04
GN 0.0004	70.80	80.32	80.06
GN 0.0005	70.80	80.34	79.99
SPN 0.1	70.72	80.30	80.11
SPN 0.2	70.83	80.30	80.08
SPN 0.3	70.76	80.10	79.80
SPN 0.4	70.61	79.77	79.51
SPN 0.5	70.44	79.55	79.31
SPKN 0.2	70.63	80.28	80.10
SPKN 0.4	70.55	80.28	80.06
SPKN 0.6	70.55	80.30	80.08
SPKN 0.8	70.51	80.35	80.06
SPKN 1.0	70.65	80.32	80.10

**Table 4:** Comparison on the McGill database between the presented algorithm and existing works.

Algorithm Name	Average Accuracy
GMI [26]	70.26%
TTKMI [26]	72.87%
TKKMI [26]	72.19%
KKKMI [26]	71.11%
TTTMI [26]	71.57%
TMI [30]	60.54%
KMI [30]	60.32%
HMI [30]	60.89%
DKMI [30]	62.01%
Ours (block size = 64×64×64, and overlap size = 16×16×16)	79.87%
Ours (block size = 32×32×32, and overlap size = 4×4×4)	80.02%
Ours (block size = 16×16×16, and overlap size = 1×1×1)	80.21%

## Data Availability

All data are available within the manuscript.

## Abbreviations

The following abbreviations are used in this manuscript:

1D	One-dimensional
2D	Two-dimensional
3D	Three-dimensional
IoT	Internet of Things
TRSI	Translation, rotation, and scale invariants
CNN	Convolutional neural networks
ANN	Artificial neural network
OM	Orthogonal moments
OP	Orthogonal polynomials
TTR	Three-term recurrence
FOBP	Fast overlapped block processing
SVM	Support vector machine
GN	Gaussian noise
SPN	Salt-and-Pepper noise
SPKN	Speckle noise
